# Ectopic Expression of Mulberry G-Proteins Alters Drought and Salt Stress Tolerance in Tobacco

**DOI:** 10.3390/ijms20010089

**Published:** 2018-12-26

**Authors:** Changying Liu, Yazhen Xu, Yang Feng, Dingpei Long, Boning Cao, Zhonghuai Xiang, Aichun Zhao

**Affiliations:** State Key Laboratory of Silkworm Genome Biology, Key Laboratory of Sericultural Biology and Genetic Breeding, Ministry of Agriculture, Southwest University, Chongqing 400716, China; lcyswu@163.com (C.L.); xuyazhen1992@163.com (Y.X.); fyzxswu@163.com (Y.F.); dplong@yeah.net (D.L.); boningcao@hotmail.com (B.C.); xbxzh@swu.edu.cn (Z.X.)

**Keywords:** mulberry, G-proteins, drought, salt stress, ROS, tobacco

## Abstract

Heterotrimeric guanine-nucleotide-binding proteins (G-proteins) play key roles in responses to various abiotic stress responses and tolerance in plants. However, the detailed mechanisms behind these roles remain unclear. Mulberry (*Morus alba* L.) can adapt to adverse abiotic stress conditions; however, little is known regarding the associated molecular mechanisms. In this study, mulberry G-protein genes, *MaGα*, *MaGβ*, *MaGγ1*, and *MaGγ2*, were independently transformed into tobacco, and the transgenic plants were used for resistance identification experiments. The ectopic expression of *MaGα* in tobacco decreased the tolerance to drought and salt stresses, while the overexpression of *MaGβ*, *MaGγ1*, and *MaGγ2* increased the tolerance. Further analysis showed that mulberry G-proteins may regulate drought and salt tolerances by modulating reactive oxygen species’ detoxification. This study revealed the roles of each mulberry G-protein subunit in abiotic stress tolerance and advances our knowledge of the molecular mechanisms underlying G-proteins’ regulation of plant abiotic stress tolerance.

## 1. Introduction

Abiotic stresses, such as drought, heat, cold, nutritional deficiency, soil salinity, and heavy metal exposure, limit plant growth, development, and reproduction [1]. To adapt to these abiotic stress conditions, plants change their physiological and biochemical processes [2,3]. Plants have evolved a series of physiological pathways, including those involved in signal recognition and transduction, transcriptional regulation, and stress responses, to tolerate stress [3]. Signal recognition and transduction play key roles in plant abiotic stress responses, and stress signal sensors, secondary messengers, receptor-like protein kinases, the Ca^2+^-signal pathway, abscisic acid (ABA) signal transduction, the salt overly sensitive (SOS) signal pathway, mitogen-activated protein kinase (MAPK) cascades, the reactive oxygen species (ROS)-related signal pathway, and transcription factors participate in this process [4,5,6,7,8,9,10,11]. 

Heterotrimeric guanine-nucleotide-binding proteins (G-proteins), a class of secondary messengers, are evolutionarily conserved signaling intermediates that regulate signal recognition and transduction, hormone perception, and immune responses [12]. G-proteins consist of Gα, Gβ, and Gγ subunits, and the Gα subunit holds a GDP and forms an inactive heterotrimeric complex with a Gβγ dimer. In the active state, the exchange of GDP for GTP on Gα results in the dissociation of G-proteins into Gα-GTP and a Gβγ dimer, and both of these components regulate downstream signaling proteins [13,14]. In animals, G-protein-coupled receptors (GPCRs) promote GDP-GTP exchange in Gα to activate the G proteins. In contrast to animals, the plant G-protein is self-activated and the regulation of G-protein signaling activation in plants occurs at the deactivation step [15]. Regulators of G-protein signaling (RGS) proteins serve as the regulatory point of G-protein activation and stimulate the rate-limiting GTPase activity of the Gα subunit [16]. Plant G-proteins are involved in many aspects of the life cycle, including morphology, seed germination, seedling development, stomatal movement, and responses to biotic and abiotic stresses in plants [17,18]. G-proteins play important roles in the responses and tolerances to abiotic stresses. Studies in Arabidopsis, rice (*Oryza sativa* L.), and maize (*Zea mays* L.) indicate that Gα negatively regulates plant salt stress tolerance and that Gβ and Gγ act as positive regulatory factors in responses to salt stress [19,20,21]. Gα and Gβ may negatively and positively, respectively, regulate drought responses, but the roles of the Gγ subunits in responses to drought require further research [22,23]. However, the roles of G-proteins in responses to other abiotic stresses (e.g., heat, cold, flooding and heavy metal exposure) remain unclear. Little is known about the molecular mechanisms involved in the plant G-protein-mediated signal pathway regulation of abiotic stress responses. 

Mulberry (*Morus alba* L.) is a deciduous and economically important perennial tree, and its leaves are the main source of food for silkworms. In addition, mulberry has multiple uses in ecology, food, and pharmaceuticals [24]. Mulberry can adapt well to adverse abiotic stresses, such as drought, flooding, water logging, high salinity, and metal exposure, but little is known regarding the molecular mechanisms involved in such processes [25]. In our previous study, we identified and characterized G-proteins-encoding genes from mulberry, and their functions in response to abiotic stresses were preliminarily analyzed [18]. Here, we transformed the genes encoding mulberry G-proteins into tobacco (*Nicotiana tabacum* L), and then assessed the changes in the drought and salt stress tolerances of the transgenic plants. The ectopic expression of *MaGα* decreased the tolerance to drought and salt stresses in tobacco, while the overexpression of *MaGβ*, *MaGγ1,* and *MaGγ2* increased the tolerances to drought and salt stresses. Furthermore, the overexpression of G-proteins could change ROS production. Overall, our results suggest a proposed pathway for G-proteins in plant tolerance to drought and salt stresses and provide a basis for further characterizations of the functions of G-proteins in abiotic stress responses and tolerances.

## 2. Results

### 2.1. Determination of Transgenic Tobacco Plants

**Figure 1:**
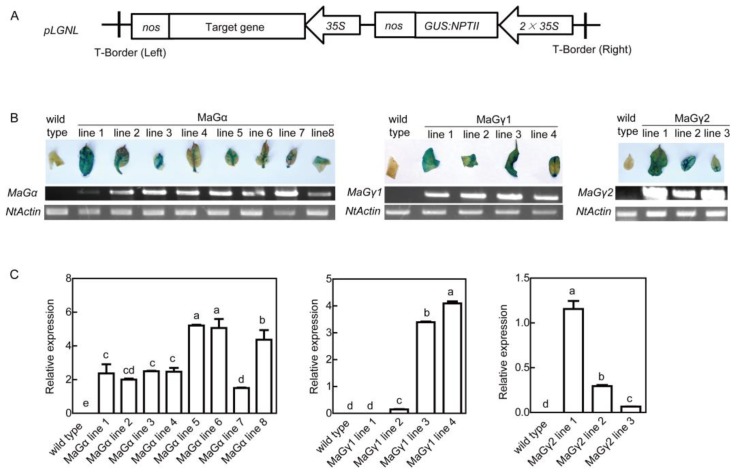
Confirmation of transgenic tobacco plants. (**A**) Diagram of the transgenic vector. (**B**) Genomic PCR analysis and histochemical GUS staining of transgenic lines. (**C**) Quantitative real-time PCR. Data are means ± SDs (*n* = 3), *P* < 0.05. Significant differences are indicated by different letters above the bars.

To evaluate the roles of mulberry G-proteins-encoding genes in plant tolerances to drought and salt stresses, the full-length sequences were independently cloned into the *pLGNL* expression vector under the control of the CaMV35S promoter and transformed into wild type (WT) tobacco plants (Figure 1A). In total, eight, four, and three independent transgenic plants overexpressing *MaGα*, *MaGγ1*, and *MaGγ2*, respectively, were obtained using β-D-glucosidase (GUS) staining and genomic PCR analyses (Figure 1B). The gene expression levels in the transgenic lines were examined by real-time quantitative reverse transcription PCR (qRT-PCR) analyses (Figure 1C). Finally, *MaGα* lines 1, 5, and 7; *MaGγ1* lines 2, 3, and 4; and *MaGγ2* lines 1, 2, and 3 were selected for further analyses. No differences in growth behavior or phenotypical traits were detected between WT and transgenic plants growing under normal conditions (Figure S1).

### 2.2. Overexpression of MaGα Reduces Plant Tolerance to Drought and Salt Stresses

**Figure 2:**
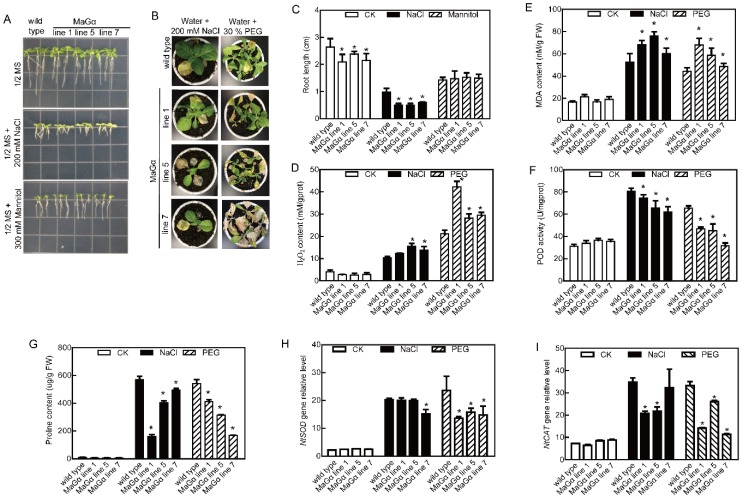
Stress tolerance analyses of *MaGα* transgenic tobacco plants. (**A**) The root growth of *MaGα* transgenic tobacco and WT plants under stress conditions. (**B**) The growth of *MaGα* transgenic tobacco and WT plants under stress conditions. (**C**) Statistical analysis of root lengths of *MaGα* transgenic tobacco and WT plants. Data are means ± SDs (*n* = 15), * *P* < 0.05. (**D**–**I**) The H_2_O_2_ content (**D**), MDA content (**E**), POD activity (**F**), proline content (**G**), and the expression levels of *NtSOD* (**H**) and *NtCAT* (**I**) in *MaGα* transgenic tobacco and WT plants under normal and stress conditions. Data are means ± SDs (*n* = 6), * *P* < 0.05.

For the root growth analysis, the 7-d-old mulberry seedlings were incubated in 1/2 MS agar medium independently containing 200 mM NaCl and 300 mM mannitol. After treatment, the roots of the *MaGα* transgenic plants were shorter than those in WT tobaccos grown under salt stress conditions, while the root lengths of transgenic and WT plants under drought stress were not significantly different (Figure 2A,C). After the five-week-old tobacco plants were treated with 200 mM NaCl and 30% polyethylene glycol (PEG) for 10 d and 14 d, respectively, the growth of WT and transgenic seedlings was significantly suppressed by both stresses, but the transgenic plants’ growth was relatively worse (Figure 2B). To understand the mechanism behind the enhanced sensitivity to drought and salt stresses caused by *MaGα* overexpression, the accumulated levels of H_2_O_2_, malondialdehyde (MDA), and proline; the peroxidase (POD) activity levels; and the expression levels of NtSOD and NtCAT were analyzed. The H_2_O_2_ and MDA contents were lower in WT tobacco plants, while the POD activity and proline content were lower in the transgenic plants grown under drought and salt stress conditions (Figure 2D–G). 3,3-diaminobenzidine (DAB) and nitro-blue tetrazolium (NBT) staining showed that the H_2_O_2_ and O^2−^ contents were greater in the leaves of *MaGα* line 7 than in WT tobacco under normal and stress conditions (Figure S2). In addition, *NtSOD* and *NtCAT* expression levels were downregulated in transgenic lines compared with those in WT plants under stress conditions (Figure 2H,I). Thus, MaGα may negatively regulate drought and salt stresses tolerance.

### 2.3. MaGβ Overexpression Increases Plant Tolerance to Salt Stress

**Figure 3:**
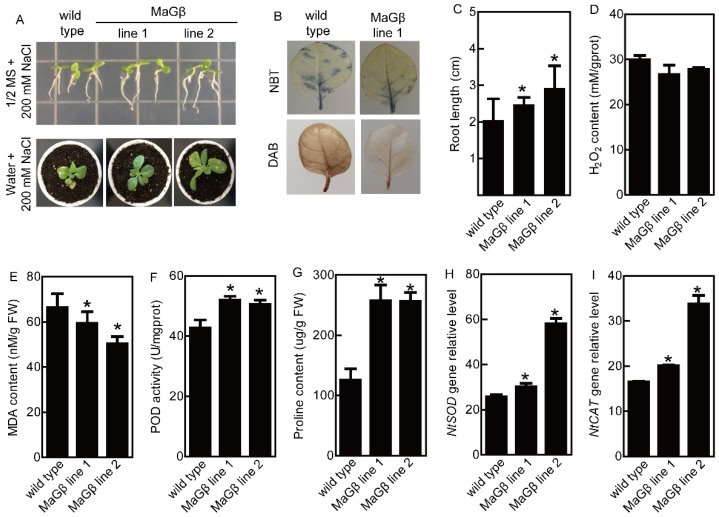
NaCl stress tolerance analyses of *MaGβ* transgenic tobacco plants. (**A**) The growth of *MaGβ* transgenic tobacco and WT plants under salt stress. (**B**) NBT and DAB staining analyses of *MaGβ* transgenic tobacco plants grown under salt stress conditions. (**C**) Statistical analysis of root lengths of *MaGβ* transgenic tobacco and WT plants. Data are means ± SDs (*n* = 15), * *P* < 0.05. (**D**–**I**) The H_2_O_2_ content (**D**), MDA content (**E**), POD activity (**F**), proline content (**G**), and the expression levels of *NtSOD* (**H**) and *NtCAT* (**I**) in *MaGβ* transgenic tobacco and WT plants under salt stress conditions. Data are means ± SDs (*n* = 6), * *P* < 0.05.

The *MaGβ* transgenic tobacco plants have been reported in our previous study [23]. The 7-d-old *MaGβ* transgenic seedlings were incubated in 1/2 MS agar medium independently containing 200 mM NaCl, and the roots of transgenic plants overexpressing *MaGβ* were longer than the roots of WT tobaccos (Figure 3A,C). After the five-week-old tobacco plants were treated with 200 mM NaCl for 14 d, the transgenic plants showed relatively better growth than WT plants (Figure 3A). The MDA contents were higher in WT tobacco plants under salt stress, while the proline content and POD activity were higher in transgenic plants (Figure 3D–G). DAB and NBT staining showed that the H_2_O_2_ and O^2−^ contents were lower in the leaves of *MaGβ* line 1 than those in WT tobacco grown under salt stress conditions (Figure 3B). In addition, *NtSOD* and *NtCAT* expression levels were upregulated in transgenic lines compared with those in WT plants under salt stress conditions (Figure 3H,I). Thus, MaGβ may positively regulate salt stress tolerance.

### 2.4. The Overexpression of MaGγ1 Increases Plant Tolerance to Drought and Salt Stresses

**Figure 4:**
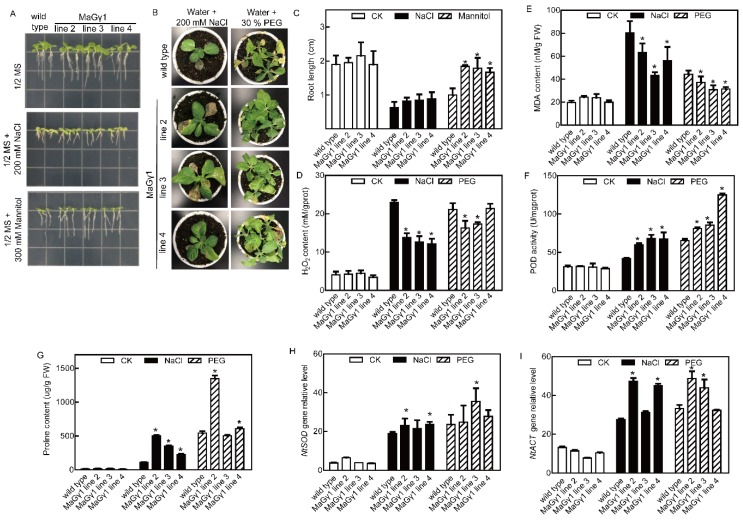
Stress tolerance analyses of *MaGγ1* transgenic tobacco plants. (**A**) The root growth of *MaGγ1* transgenic tobacco and WT plants under stress conditions. (**B**) The growth of *MaGγ1* transgenic tobacco and WT plants under stress conditions. (**C**) Statistical analysis of root lengths of *MaGγ1* transgenic tobacco and WT plants. Data are means ± SDs (*n* = 15), * *P* < 0.05. (**D**–**I**) The H_2_O_2_ content (**D**), MDA content (**E**), POD activity (**F**), proline content (**G**), and the expression levels of *NtSOD* (**H**) and *NtCAT* (**I**) in *MaGγ1* transgenic tobacco and WT plants under normal and stress conditions. Data are means ± SDs (*n* = 6), * *P* < 0.05.

The 7-d-old seedlings were incubated in 1/2 MS agar medium independently containing 200 mM NaCl and 300 mM mannitol, and the roots of transgenic plants overexpressing *MaGγ1* were longer than the WT roots under drought stress, while the lengths of transgenic and WT roots were not significantly different under salt stress condition (Figure 4A,C). After the five-week-old tobacco plants were independently treated with 200 mM NaCl and 30% PEG for 25 d and 14 d, respectively, the transgenic plants showed relatively better growth than WT plants (Figure 4B). The H_2_O_2_ and MDA contents were higher in WT tobacco plants subjected to drought and salt stresses, while the proline content and POD activity were higher in the transgenic plants (Figure 4D–G). The results of DAB and NBT staining showed that the H_2_O_2_ and O^2−^ contents were lower in the leaves of *MaGγ1* line 3 than those in WT tobacco under normal and stress conditions (Figure S2). In addition, *NtSOD* and *NtCAT* expression levels were upregulated in transgenic lines compared with those in WT plants grown under stress conditions (Figure 4H,I). Thus, MaGγ1 may positively regulate drought and salt stress tolerance. 

### 2.5. The Overexpression of MaGγ2 Increases Plant Tolerance to Drought and Salt Stresses

**Figure 5:**
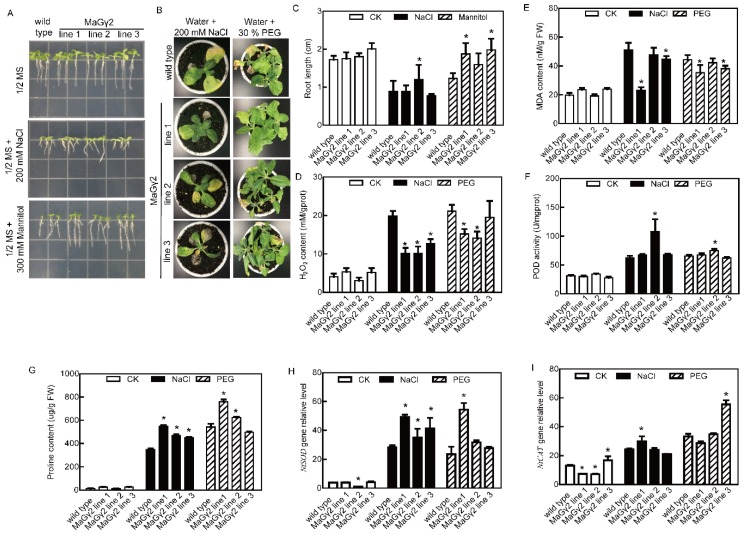
Stress tolerance analyses of *MaGγ2* transgenic tobacco plants. (**A**) The root growth of *MaGγ2* transgenic tobacco and WT plants under stress conditions. (**B**) The growth of *MaGγ2* transgenic tobacco and WT plants under stress conditions. (**C**) Statistical analysis of root lengths of *MaGγ2* transgenic tobacco and WT plants. Data are means ± SDs (*n* = 15), * *P* < 0.05. (**D**–**I**) The H_2_O_2_ content (**D**), MDA content (**E**), POD activity (**F**), proline content (**G**), and the expression levels of *NtSOD* (**H**) and *NtCAT* (**I**) in *MaGγ2* transgenic tobacco and WT plants under normal and stress conditions. Data are means ± SDs (*n* = 6), * *P* < 0.05.

The 7-d-old seedlings were incubated in 1/2 MS agar medium independently containing 200 mM NaCl and 300 mM mannitol, and the roots of transgenic plants overexpressing *MaGγ2* were longer than the WT roots under drought and salt stress conditions (Figure 5A,C). After the five-week-old tobacco plants were independently treated with 200 mM NaCl and 30% PEG for 25 d and 14 d, respectively, the transgenic plants showed relatively better growth than WT plants (Figure 5B). The H_2_O_2_ and MDA contents were higher in WT tobacco plants, while the POD activity and proline content were higher in the transgenic plants grown under drought and salt stresses (Figure 5D–G). DAB and NBT staining showed that the H_2_O_2_ and O^2−^ contents were lower in the leaves of *MaGγ2* line 1 than those in WT tobacco under normal and stress conditions (Figure S2). In addition, *NtSOD* and *NtCAT* expression levels were upregulated in transgenic lines compared with those in WT plants under stress conditions (Figure 5H,I). Thus, MaGγ2 may positively regulate drought and salt stress tolerance.

## 3. Discussion

G-proteins play important roles in responses to abiotic stresses, especially drought and high salinity. However, the roles of each G-protein subunit in plant responses to drought and salt stresses are not fully understood. Loss-of-function analyses of Arabidopsis mutants suggested that the three subunits of G-proteins had different roles in salt stress tolerance, namely, Gα has a negative role in salt stress responses and Gβ and Gγ act as positive factors [19]. The functions of rice and maize Gα subunits and rice Gγ subunit RGG1 are similar to those in Arabidopsis [20,21]. In this study, mulberry G-protein genes *MaGα*, *MaGβ*, *MaGγ1*, and *MaGγ2* were independently transformed into tobacco, and the transgenic plants were used for resistance identification experiments. *MaGα* negatively regulated salt stress responses, and *MaGβ*, *MaGγ1*, and *MaGγ2* positively regulated salt stress responses (Figure 2, Figure 3, Figure 4 and Figure 5), which was similar to results from Arabidopsis, rice, and maize [19,20,21,26] and indicates a conservation of G-protein roles in responses to salt stress in various plants. However, the roles of G-proteins in responses to drought stress are not yet fully clear. The overexpression of *MaGα* decreased the tolerance to drought, which is similar to the results in rice and apple (*Malus domestica* Borkh.) [22,27]. There have been no reports on the roles of G-protein γ subunits in plant tolerance to abiotic stresses. Here, the overexpression of *MaGγ1* and *MaGγ2* increased plant tolerance to drought stress. Additionally, our previous study indicated that mulberry and Arabidopsis G-protein β subunits positively regulate drought tolerance [23]. Thus, the roles of G-proteins in plant drought tolerance are the same as those in salt stress tolerance. Therefore, we conclude that plant Gα negatively regulates plant drought and salt stress tolerance, while Gβ and Gγ act as positive regulatory factors in drought and salt stress tolerance (Table 1).

Plants often suffer from a variety of abiotic stresses throughout their plant growth and development [28]. ROS is one of versatile signaling molecules that play an important role in abiotic stress responses and tolerances [29]. During the early response to abiotic stresses, plants rapidly generate ROS molecules and activate downstream stress responsive signaling pathways [29]. However, typically, abiotic stresses, such as drought, heat, and salinity, result in the excessive accumulation of ROS, which causes oxidative damage to membranes, proteins, and RNA and DNA molecules, and eventually leads to the oxidative destruction of the cell [10]. To maintain cellular homeostasis, the ROS scavenging enzymes SOD, glutathione peroxidase, POD, ascorbate peroxidase, and catalase participate in protecting plant cells from ROS toxicity caused by abiotic stresses [30]. Previous studies suggested that G-proteins may regulate ROS production in Arabidopsis guard cells, and the loss of AtGPA1 interrupts ROS production [31,32]. The overexpression of the rice G-protein γ subunit *RGG1* enhances tolerance to salt stress by elevating ROS detoxification [20]. The ectopic expression of mulberry MaGβ in tobacco enhances tolerance to drought stress by promoting the alleviation of ROS accumulation [23]. In this study, *MaGα*’s overexpression increased the H_2_O_2_ and O^2−^ contents under drought and salt stress conditions, while *MaGβ*, *MaGγ1*, and *MaGγ2* overexpression decreased the accumulation of ROS. Additionally, *MaGα*’s overexpression reduced the activity of POD and the expression levels of *NtSOD* and *NtCAT* under drought and salt stress conditions, while the overexpression of *MaGβ*, *MaGγ1*, and *MaGγ2* increased the activities of POD and the expression levels of antioxidant genes. Therefore, G-proteins may regulate plant drought and salt stresses responses by modulating the detoxification of ROS. How do G-proteins regulate ROS production in response to abiotic stresses? We proposed that G-proteins may affect ROS production by regulating the activities and gene expression of antioxidant proteins through interaction or intermediary signal pathways. A gene ontology enrichment analysis of the Arabidopsis G-protein interactome showed that some effector proteins respond to abiotic stresses, such as salt stress, osmotic stress, cold, and cadmium ion exposure [17,19]. The interacting proteins of Arabidopsis AtAGB1 include several proteins that play important roles in abiotic stress tolerances [23]. The interacting partners of Gβ in pea (*Pisum sativum* L.) and *Brassica juncea* included several important stress-responsive proteins, such as pea thioredoxin H, *B. juncea* thioredoxin-M2, and 2-cys-peroxiredoxin XA, which are key components involved in the ROS-scavenging pathway [33,34]. In rice, the G-protein γ subunit RGG1 interacts with different stress-responsive proteins that play active roles in stress responses, including a calcium/calmodulin-dependent protein kinase that regulates the production of ROS under salinity stress [20]. A recent study suggested that Arabidopsis G-protein β subunit AGB1 is involved in the RALF-FERONIA regulation of stomatal movement by regulating ABA signal and Ca^2+^ production [35]. AGB1 and FERONIA may act synergistically in salt tolerance by regulating ROS production and K^+^ net uptake [36]. Additionally, Arabidopsis G-protein subunits can physically interact with the MAPK cascade proteins (e.g., AtMPK3, AtMPK6, AtMKK4, and AtMKK5) that play important roles in biotic and abiotic stress responses in plants [37]. However, the exact mechanisms of G-proteins involved in abiotic stress tolerance and ROS detoxification require investigation. Meanwhile, identification and function analysis of the intermediate signaling elements of G-proteins involved in ROS detoxification in mulberry needs to be conducted. 

In conclusion, the roles of the mulberry G-proteins in responses to drought and salt stresses were revealed. MaGα acts as a negative factor in plant drought and salt stress tolerance, while Gβ and Gγ positively regulate drought and salt stress tolerance. In addition, mulberry G-proteins regulate drought and salt tolerances by modulating ROS detoxification. However, more studies are needed to elucidate the mechanisms of G-proteins in response to abiotic stresses. Overall, our data will help improve the understanding of G-protein-mediated abiotic stress tolerance in plants. The present study also provides a basis for further characterizations of the functions of G-proteins from mulberry and other plants in abiotic stress tolerances.

## 4. Materials and Methods

### 4.1. Plasmid Construction and Plant Transformation

The full-length coding sequences of *MaGγ1* (KX099866) and *MaGγ2* (KX099867) from mulberry were independently cloned into the SalI and EcoRI restriction sites of the pLGNL expression vector under the control of the CaMV35S promoter, and the *MaGα* (KX099864) genes were independently cloned into the KpnI and EcoRI restriction sites of the *pLGNL* expression vector. The recombinant plasmids were transformed into *Agrobacterium tumefaciens* strain GV3101. The positive *A. tumefaciens* harboring the transgenic plasmids were transformed into tobacco (K326) plants using the leaf disc method. The methods of transformation and identification of transgenic plants were similar to those described in a previous study [38]. Transgenic tobacco seeds were screened on 1/2 MS medium containing a final concentration of 50 mg/L kanamycin, and the grown seedlings were then confirmed by GUS staining and genomic PCR. Additionally, the expression levels of exogenous genes in transgenic tobacco plants were detected by qRT-PCR analysis. The primers for these experiments are shown in Table S1.

### 4.2. Stress Tolerance Analysis of Transgenic Plants

The stress treatment methods and measurements of physiological parameters were similar to those described in our previous study [23]. For the root growth analysis, the 7-d-old seedlings were incubated in 1/2 MS agar medium independently supplemented with 200 mM NaCl and 300 mM mannitol for 7 d, and the root growth was recorded. Each treatment was replicated in three plates. The transgenic lines and WT tobacco plants were grown on 1/2 MS medium and then transferred into soil in a climate chamber (24 °C, 16-h light/8-h dark photoperiod). The five-week-old plants were independently irrigated with water containing 30% PEG and 200 mM NaCl until significant differences appeared between the transgenic lines and WT tobacco. Each treatment was replicated three times. After stress treatments, the shoots of the treated plants were collected, and the MDA, H_2_O_2_, and proline contents, as well as the POD activity, were measured using their respective test kits (Jiancheng Bioengineering Institute, Nanjing, China) according to the manufacturer’s instructions. Three plants were used per sample, and each sample was measured six times. *NtSOD* and *NtCAT* expression levels of treated plants were analyzed by qRT-PCR. In addition, the three-week-old tobacco seedlings were independently incubated with 100 mM NaCl and 20% PEG for 24 h and used for DAB and NBT staining.

### 4.3. Quantitative Real-Time PCR

The treated tobacco plants were collected for total RNA extraction using TRIzol reagent (Invitrogen, Carlsbad, CA, USA). The first-strand cDNA synthesis and qRT-PCR analysis were performed as described in a previous study [39]. The first-strand cDNAs were synthesized using the Perfect Real Time version of the PrimerScript™ RT reagent Kit with gDNA Eraser (Perfect Real Time) (TaKaRa, Dalian, China). Ten-fold diluted cDNA was used in the qRT-PCR. The qRT-PCR reactions were performed using the Applied Biosystems StepOne Plus Real-Time PCR System (Applied Biosystems, Foster City, CA, USA). *NtActin* (U60489) was used as the internal control, and the relative expression was defined as 2^−[*C*t(target gene) Ct(control gene)]^. All qRT-PCRs were performed with at least three independent biological replicates. The primers used are specified in Table S1.

### 4.4. Statistical Analyses

The results were organized and analyzed by Excel 2013 (Microsoft, Redmond, WA, USA) and GraphPad Prism 6.0 (GraphPad Software, La Jolla, USA). The results are presented as mean values ± SDs. The significant differences between samples were analyzed using a one-way ANOVA in SPSS Statistics 17.0 (SPSS Inc., Chicago, IL, USA). Comparisons among means were conducted using Duncan’s test calculated at *P* < 0.05. Mean values that were significantly different from each other were indicated by asterisks or different letters. 

## Figures and Tables

**Table 1:** The roles of G-proteins from different species in plant tolerance to drought and salt stresses.

	Gα	Gβ	Gγ
**Salt stress:**			
*A. thaliana*	↓	**↑**	**↑**
*O. sativa*	↓	—	**↑**
*Z. mays*	↓	—	—
*M. alba*	↓	**↑**	**↑**
**Drought:**			
*A. thaliana*	—	**↑**	—
*O. sativa*	↓	**↑**	—
*M. domestica*	↓	—	—
*M. alba*	↓	**↑**	**↑**

The *up* and *down arrows* represent positive and negative roles in response to stress conditions, respectively. The *line* indicates no reports on the role in response to stress conditions.

## Supplementary Materials

Supplementary materials can be found at http://www.mdpi.com/1422-0067/20/1/89/s1.

## Abbreviations

DAB	3, 3-diaminobenzidine
Gα	G-protein α subunit
Gβ	G-protein β subunit
Gγ	G-protein γ subunit
G-proteins	heterotrimeric guanine-nucleotide-binding proteins
MDA	malondialdehyde
NBT	nitro-blue tetrazolium
PEG	polyethylene glycol
POD	peroxidase
qRT-PCR	reverse transcription quantitative PCR
ROS	reactive oxygen species
WT	wild type
