# Supplementary material for: Ectopic Expression of Mulberry G-Proteins Alters Drought and Salt Stress Tolerance in Tobacco
Source: Int J Mol Sci. 2018 Dec 26;20(1):89. doi: 10.3390/ijms20010089 (PMC6337368; doi:10.3390/ijms20010089)
Supplement: Supplementary file 1 [file ijms-20-00089-s001.pdf]

Table S1. Oligo DNA sequence for PCR primers.

Purpose	Primer name	Sequence (5'-3')
Plant expression vectors construction	MaGα F	GGGGTACCATGGGCTTACTATGCAGCAGGA
	MaGα R	GGAATTCTCACAACAAGCCAGCCTCGAAG
	MaGγ1 F	ACGCGTCGACATGGAGCCCGAAACGGCATCGT
	MaGγ1 R	GGAATTCTCAGAGTATGACACACCTACAG
	MaGγ2 F	ACGCGTCGACATGCAATCGAGTGGGTCCCAAT
	MaGγ2 R	GGAATTCTCACAGGATCCAGCATCTGCAG
Expression pattern analysis	NtSOD F	AGCTACATGACGCCATTTCC
	NtSOD R	CCCTGTAAAGCAGCACCTTC
	NtCAT F	AGGTACCGCTCATTCACACC
	NtCAT R	AAGCAAGCTTTTGACCCAGA
	NtActin F	TCACAGAAGCTCCTCCTAATCCA
	NtActin R	GAGGGAAAGAACAGCCTGAATG

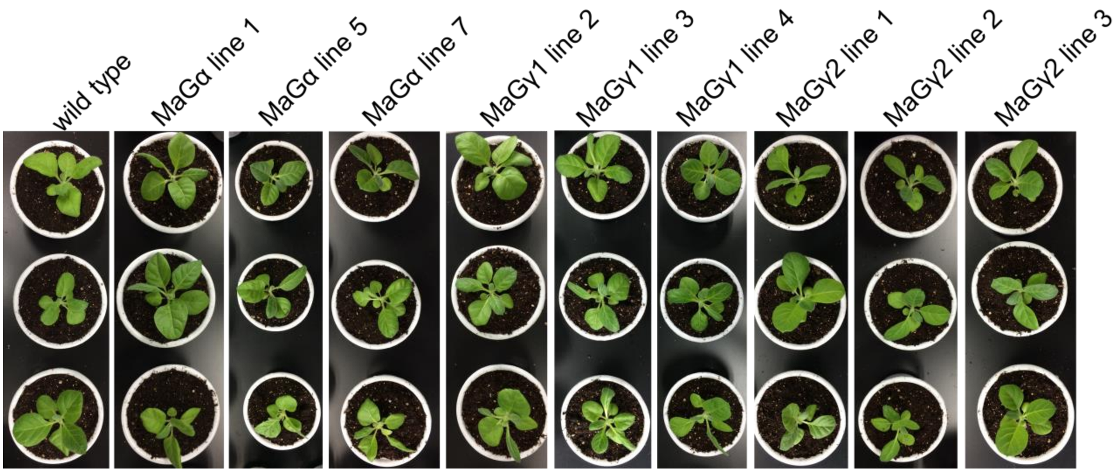

Figure S1. The growth of transgenic tobacco and WT plants under normal conditions.

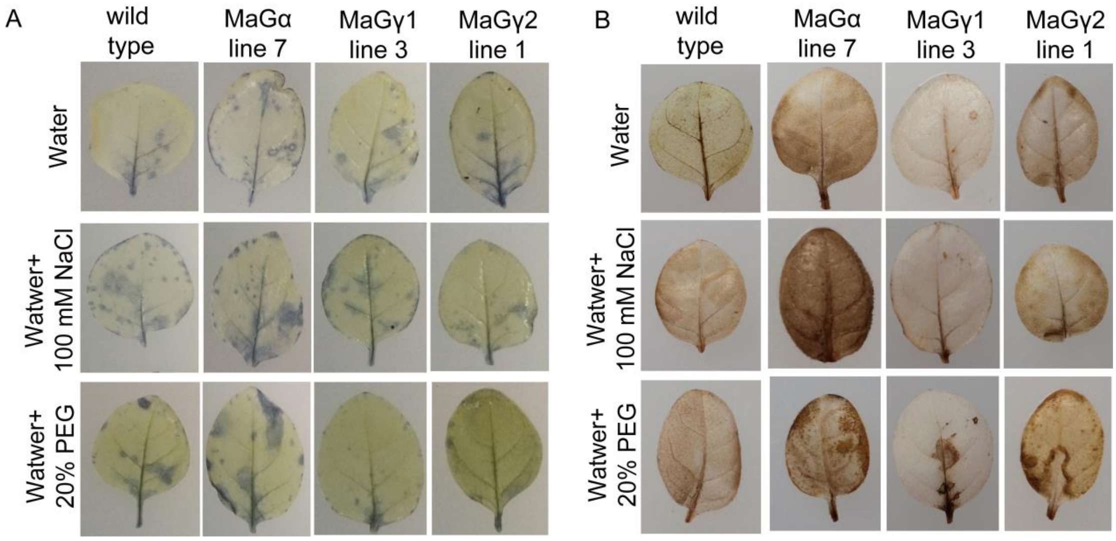

Figure S2. NBT and DAB staining analyses of transgenic tobaccos. NBT staining (**A**) and DAB staining (**B**) of transgenic tobacco leaves under normal and stress conditions.
